# Effects of Purple Corn Anthocyanin on Growth Performance, Meat Quality, Muscle Antioxidant Status, and Fatty Acid Profiles in Goats

**DOI:** 10.3390/foods11091255

**Published:** 2022-04-27

**Authors:** Xingzhou Tian, Jiaxuan Li, Qingyuan Luo, Xu Wang, Tiansong Wang, Di Zhou, Lingling Xie, Chao Ban, Qi Lu

**Affiliations:** 1 Key Laboratory of Animal Genetics, Breeding and Reproduction in the Plateau Mountainous Region, Ministry of Education, College of Animal Science, Guizhou University, Guiyang 550025, China; tianxingzhou@yeah.net (X.T.); jiaxuanli8899@163.com (J.L.); natty579@163.com (Q.L.); wx12345678888@163.com (X.W.); 18285123904@163.com (C.B.); 2 School of Agronomy, Tongren Polytechnic College, Tongren 554300, China; wangtiansong9716@yeah.net; 3 Testing Center for Livestock and Poultry Germplasm, Guizhou Agricultural and Rural Affairs Office, Guiyang 550018, China; dizhougz@163.com (D.Z.); snynctxll@163.com (L.X.)

**Keywords:** anthocyanin, antioxidant activity, fatty acid profile, gene expression, goat, meat quality

## Abstract

**Simple Summary:**

Currently, unsaturated fatty acids (UFAs) in goat meat have gained popularity among consumers based on the benefits to human health. However, UFAs are prone to lipid oxidation. Purple corn is rich in anthocyanins, which have a wide range of biological activities, such as antioxidation, scavenging free radicals, and preventing lipid peroxidation. This study hypothesized that purple corn anthocyanin can improve meat quality and prevent lipid oxidation by promoting antioxidant status and UFA levels in *longissimus thoracis et lumborum* muscle of growing goats. The results of the current study indicate that dietary supplementation with purple corn anthocyanins has the potential to improve meat quality and prevent lipid oxidation by promoting antioxidant status and regulating related antioxidant gene expression, as well as improving polyunsaturated fatty acid concentrations in the *longissimus thoracis et lumborum* muscle of growing goats.

**Abstract:**

This study was conducted to examine the effect of purple corn anthocyanin on performance, meat quality, muscle antioxidant activity, antioxidant gene expression, and fatty acid profiles in goats. The feeding trial period lasted 74 d. The adaptation period was 14 d, and the formal experimental period was 60 d. Eighteen Qianbei-pockmarked goats (Guizhou native goat breed; body weight, 21.38 ± 1.61 kg; mean ± standard deviation) were randomly allotted into three equal groups, including a control with no purple corn pigment (PCP) and groups receiving either 0.5 g/d PCP or 1.0 g/d PCP. The inclusion of PCP did not affect (*p* > 0.05) the dry matter intake, average daily gain, or feed conversion ratio compared to the control group. The addition of PCP reduced (*p* < 0.05) shear force in the *longissimus thoracis et lumborum* muscle (LTL) during the growth phase of the goats. Goats receiving PCP showed higher (*p* < 0.05) levels of reduced glutathione, 2,2-diphenyl-1-picrylhydrazyl scavenging activity and peroxidase in LTL compared to the control. Moreover, compared to the control, the PCP group displayed lower (*p* < 0.05) concentrations of 12:0, C16:0, and total saturated fatty acids, but increased (*p* < 0.05) concentrations of various unsaturated fatty acids, including C18:1n9, C20:3n6, C20:4n6, C18:2n6 *cis*, C20:3n6, C22:5n3, C22:6n3, and total polyunsaturated fatty acids (PUFAs). The abundance of nuclear factor, erythroid 2 like 2, superoxide dismutase 1, glutathione peroxidase 1, and catalase was upregulated (*p* < 0.05) in the LTL of goats receiving 0.5 g/d PCP in comparison to the other groups. Collectively, result of the current study indicated that PCP anthocyanin could be used as a source of natural functional additive because anthocyanin-rich PCP has the potential to improve meat quality and enhance muscle antioxidant status as well as improve the proportions of PUFAs in goat muscle.

## 1. Introduction

Consumers are increasingly interested in obtaining high-quality meat products with good nutritional profiles and health value [1]. Ruminant meat has a high saturated fatty acid (SFA) content because of rumen biohydrogenation [2]. Specifically, polyunsaturated fatty acid (PUFA) compounds are essential to strengthening the body’s immune system and improving the health of consumers. However, high concentrations of PUFAs in goat meat are often responsible for the reduction in oxidative stability because of lipid oxidation, leading to the formation of reactive oxygen species (ROS) and free radicals (FRs), thereby reducing product shelf life and causing various diseases, such as cancer and cardiovascular diseases [3].

Dietary supplementation of antioxidants can be transferred to the muscle, where they work with natural defence systems and thus counteract pro-oxidants [4]. Although synthetic antioxidants have been widely used industrially to control lipid oxidation in muscle, consumer concern over their safety and toxicity has initiated a search for natural sources of antioxidants [5]. For this reason, there is growing interest in natural antioxidants, especially phenolics and flavonoids, which are safe and bioactive [3]. Previous studies have demonstrated that dietary natural antioxidant extracts in ruminants may be an effective approach for improving antioxidant potential and alleviating lipid oxidation, and thus they have become popular with numerous consumers [6]. Additionally, Yakan et al. [7] showed that adding natural antioxidants to Damascus male kid diets can increase the percentage of total unsaturated fatty acids (UFAs) and omega-3 PUFAs and decrease the percentage of SFAs in muscle.

Anthocyanins are large polyphenol compounds that have strong natural antioxidation properties and widely exist in plants and food [8]. In addition, anthocyanins are suitable sources of natural active substances for ruminants and impart health benefits, including scavenging FRs and improving the body’s antioxidant capacity [9]. Anthocyanins can impact blood lipid parameters by regulating related gene expression in the muscle of goats [10]. Dietary anthocyanins can improve rumen fermentation parameters and change the ruminal microbiota in goats [11]. In other words, anthocyanins may protect PUFAs by modulating lipid mechanisms and ruminal biohydrogenation, thereby improving PUFA levels in ruminant meat. Supplementation of anthocyanins in sheep feed could alleviate oxidative stress (OS) and help prevent PUFA loss, thereby significantly improving PUFA concentrations [12]. Prommachart et al. [13] demonstrated that the feeding of anthocyanin-extracted residue could reduce meat oxidation and increase omega-3 PUFA concentration, resulting in the extension of the shelf life of the meat of male dairy cattle. 

Additionally, we previously confirmed that purple corn pigment (PCP) could maintain PUFA levels in milk during the storage period [14,15]. Hence, anthocyanins can donate electrons to FRs with unpaired electrons and reduce agents in the electron-transfer reaction pathway in ruminant meat, inserting additional unsaturated bonds into already existing PUFAs [13]. Despite these studies, the current understanding of the effect of anthocyanins from PCP on meat quality, muscle antioxidant activity, and fatty acid (FA) profiles in goats is limited. Here, we hypothesize that the inclusion of PCP could improve meat quality and enhance the antioxidative ability as well as PUFA concentration in goat meat. Accordingly, the present study was conducted to examine the effects of PCP anthocyanins on growth performance, meat quality, antioxidant status, FA content, and gene expression of *longissimus thoracis et lumborum* muscle (LTL) in goats.

## 2. Materials and Methods

### 2.1. Materials

The PCP was purchased from Nanjing Herd Source Biotechnology Co., Ltd. (Nanjing, China). The anthocyanin composition of PCP extracted using 1.5 M HCl dissolved in a 95% ethanol solution (ethanol:HCl = 85:15) was determined by high performance liquid chromatography tandem mass spectrometry, according to Tian et al. [14]. The anthocyanin compositions of pelargonidin (Pel), peonidin (Peo), cyanidin (Cya), malvidin (Mal), petunidin (Pet), and delphinidin (Del) were determined. The total anthocyanin content was calculated from the following equation: TA = Pel + Peo + Cya + Mal + Pet + Del. The anthocyanin composition of commercial PCP is listed in Table 1.

### 2.2. Animals, Diets, and Experimental Design

The feeding trial was conducted at Fuxing Husbandry Co., Ltd., Guizhou, China (106.198244 E, 28.26403 N). The feeding trial period lasted 74 d. The adaptation period was 14 d. Thus, the formal experimental period was 60 d. Eighteen Qianbei-pockmarked wether goats (Guizhou native goat breed; age, 90 d; body weight (BW), 21.38 ± 1.61 kg; mean ± standard deviation) were randomly divided into three groups, including a control with no PCP (CON) and treatments receiving either 0.5 g/d PCP (LA) or 1.0 g/d PCP (HA), using a completely randomized design. The Qianbei-pockmarked goat is one of main goat breeds in the Guizhou province in China and has strong tolerance to the inclement environment, roughage resistance, good meat quality, and has deep academic research and practical application value. The level of PCP used in this study was based on Tian et al. [16]. Briefly, the control group goats were fed a basal diet, and 0.5 g and 1.0 g/d PCP were added to the diets of treatments 1 and 2, respectively. The concentrate, premix, and PCP were mixed first, and then they were added to roughage to prepare the total mixed ration (Table 2). The nutrient requirements of the experimental animals were based on the National Research Council [17]. All of the experimental kids were housed in clean individual pens, and water was provided freely during the entire experimental period. Equal rations were provided twice daily at 08:30 and 16:30 for *ad libitum* intake and 10% refusals on an as-fed basis.

### 2.3. Chemical Composition

Approximately 100 g of experimental diet was collected once weekly and stored at −20 °C. After the end of the feeding trials, the basal diet was dried at 65 °C in a vacuum oven for 72 h, ground, and passed through a 1-mm sieve. Dry matter, crude protein, ether extract, ash, calcium, and phosphorus were measured as described in the Association of Official Analytical Chemists [18]. Neutral detergent fibre (NDF) and acid detergent fibre (ADF) were determined according to Van Soest et al. [19] using an FT 122 Fibertec^TM^ analyser (Foss, Hillerød, Denmark). Organic matter and hemicellulose were obtained by 100 minus the ash and NDF minus ADF, respectively [20]. Gross energy was detected using a calorimeter (WGR-WR3, Changsha BENTE Instrument Co., Ltd., Changsha, China). Each sample was run in triplicate.

### 2.4. Growth Performance

Dry matter intake (DMI) was calculated daily throughout the entire study period. The BW of each kid was weighed on the 1st day and 74th day of the trial period before morning feeding. The body weight change (BWC), average daily gain (ADG), and feed conversion ratio (FCR) were calculated until the end of the experiment, according to Biazen et al. [21]. The formulas for calculation were as follows: BWC (kg) = final weight (kg) − initial weight (kg); ADG (g/d) = BWC (kg)/74 × 1000; FCR = DMI (g/d)/ADG (g/d).

### 2.5. Meat Quality

All animals were slaughtered at the end of the experiment, and carcasses were processed as described by Danforth [22]. The left LTL of the kids was separated for assessment of meat quality. The pH of the LTL was measured at 45 min and 24 h (put into a chiller at 4 °C) after slaughter by a pH-star (Matthäus, Eckelsheim, Germany). The pH value was updated twice per second until the value was stable, and the data were recorded. Each sample per time point was run in triplicate. To determine the rate of water loss, a 1-cm thick sample was cut using a 2.5-cm diameter with a 5 cm^2^ area circular sampler, and the pre-pressure weight was determined. Next, the sample was sandwiched between two layers of gauze and placed on a platform with 18 layers of qualitative medium-speed filter paper above and below, and subjected to 35 kg of pressure for 5 min. The platform was removed, and the meat sample was immediately peeled off from the gauze and weighed to determine the post-pressure weight. The percentage of water loss was calculated using the following equation: Percentage of water loss (%) = [(pre-pressure weight − post-pressure weight)/pre-pressure weight] × 100. For drip loss, approximately 10 g and 3-cm length cubes of LTL were manually trimmed, weighed, suspended in a fishhook in an inflated plastic bag, sealed, and stored for 24 h at 4 °C. Each sample was weighed after removing the fishhook and dried on filter paper. Drip loss was calculated as follows: Drip loss (%) = [(initial weight − final weight)/initial weight] × 100. For cooking loss, approximately 100 g of LTL was weighed without fascia, epimysium, or fat. The sample was placed in a steamer and cooked for 30 min, and then the steam meat sample was weighed after cooling at 4 °C for 2 h. Cooking loss was calculated as follows: Cooking loss (%) = (fresh weight − cooked weight)/fresh weight × 100. Meat colour (Opto), defined as the L-value (L*) in the Cielab system, was detected at 45 min after slaughter using Opto-Star equipment (Company MATTHÄU KLAUSA, Eckelsheim, Germany). The shear force of the sample cores was measured perpendicularly to the direction of the muscle fibre using a digital display tenderness meter (Xielikeji Co., Ltd., Harbin, China) with a load cell of 15 kg and a 200-mm/min crosshead speed. Each index was run in three replicates.

### 2.6. Antioxidant Activity

The muscle sample was washed with precooled phosphate buffered solution (PBS; 0.01 M, pH = 7.4) to remove residual blood, weighed, and then cut up. The chopped tissues and PBS were added to a glass homogenizer at a ratio of 1:9 and thoroughly ground on ice. The homogenate was ultrasonicated using a Bransonic^®^ ultrasonic cleaner (Branson Ultrasonics Corp., Danbury, CT, USA). The homogenate was centrifuged at 4000× *g* for 10 min at 4 °C, and the supernatant was transferred to a 1.5-mL tube and stored at −80 °C.

Total antioxidant capacity (TAC), superoxide dismutase (SOD), glutathione peroxidase (GPX), reduced glutathione (GSH), catalase (CAT), peroxidase (POD), malondialdehyde (MDA), nitric oxide (NO), superoxide anion (O_2_^−^), and hydroxyl FR (OH) were determined using commercially available kits from Nanjing Jiangcheng Bioengineering Institute (Nanjing, China; Product codes A015-1, A001-3, A005, A006-1-1, A007-1-1, A084-3-1, A084-3-1, A012-1, A052-1-1, and A018-1-1, respectively). The above parameters were detected by spectrophotometry, and the absorbance was analysed using a microplate reader (Epoch, BioTek, Luzern, Switzerland). All of the measurement procedures strictly followed the manufacturers’ recommendations. An FR parameter of 2,2-diphenyl-1-picrylhydrazyl (DPPH; Product code: 101845869, Sigma–Aldrich, St. Louis, MO, USA) scavenging activity was measured by spectrophotometry [23]. Briefly, a 20 μL aliquot of each sample was mixed with 0.6 mL of 0.1 mmol/L DPPH solution in a 1.5-mL tube. The mixture was centrifuged at 4000× *g* for 10 min at 4 °C, and then 200 μL of the supernatant was immediately transferred into a 96-well plate (TCP011096, JET-BIOFIL^®^, Beiden Biological Technology Co., Ltd., Nanjing, China) and incubated in the dark at 37 °C for 30 min. The absorbance was analysed at a wavelength of 517 nm using a microplate reader (Epoch, BioTek, Luzern, Switzerland). DPPH scavenging activity was calculated using the following equation: DPPH scavenging activity (%) = (Ac − As) × 100/Ac, where Ac is the absorbance of the control and As is the absorbance of the sample.

### 2.7. Fatty Acid Profiles

The FAs were extracted from the LTL according to the Chinese standard GB 5009.168-2016 [24], with a minor modification. Briefly, approximately 100 mg of each LTL sample was placed into a 5-mL tube, to which 3 mL of chloroform-methanol solution (2:1) and two steel balls were added, and the mixture was shaken vigorously using a TissueLyser at 60 Hz for 10 min. Then, ultrasonic extraction was added to 0.6 mL normal saline and was performed at room temperature for 30 min, followed by centrifugation at 3500× *g* at 4 °C for 10 min (H1850R, Hunan Xiangyi Centrifuge Instrument Co., Ltd., Changsha, China), and the supernatant was transferred to a 2-mL centrifuge tube. The sample was esterified by 0.8 mL of 2% sodium hydroxide-methanol solution, and a reflux condenser was connected. After cooling to room temperature, 1 mL n-heptane was added, and the tube was thoroughly mixed, left to stand for 5 min, and centrifuged at 10,000× *g* at 4 °C for 5 min. Then, 100 mg of anhydrous sodium sulfate powder was added after the supernatant was transferred to a 2-mL tube with sufficient mixing and left to stand for 5 min, and the supernatant was transferred to a 1.5-mL tube and stored at −20 °C until measurement. Individual FAs were detected using gas chromatography (GC; Agilent 6890, Agilent Technologies, Santa Clara, CA, USA). The GC conditions were as follows: separation of FA by a strong polar stationary phase of a polydicyanopropyl siloxane capillary column (100 m × 0.25 mm × 0.20 µm), the injection volume was 1 µL, the injection port temperature was 270 °C, and the detector temperature was 280 °C. The temperature program included: an initial temperature of 100 °C for 13 min with a 10 °C/min rise to 180 °C, and this temperature was held for 6 min, a 1 °C/min rise to 200 °C, and this temperature was held for 20 min, then, a 4 °C/min rise to 230 °C, and this temperature was held for 10.5 min. The split ratio was 100:1, and nitrogen gas was used as the carrier. Individual FAs were detected from the chromatogram peak areas, and the data were expressed in grams per 100 g of FAs.

### 2.8. Gene Expression

The total RNA was extracted as follows: First, 100 mg of each LTL sample was weighed and moved into a 1.5-mL sterilized tube, 1 mL of RNA extraction solution (Cat. No. G3013; Wuhan Servicebio Technology Co., Ltd., Wuhan, China) was added, and the tube was shaken vigorously before running in a homogenizer. Second, the total RNA was dissolved with HyPureTM Molecular Biology Grade Water. Third, the RNA concentration and purity were measured using a NanoDrop 2000 machine (Thermo Fisher Scientific, Waltham, MA, USA). Complementary deoxyribonucleic acid (cDNA) synthesis was determined by a commercial cDNA synthesis kit (Cat. No. G3330; Wuhan Servicebio Technology Co., Ltd., Wuhan, China).

As shown in Table 3, in this study, a total of four target genes were detected, including nuclear factor, erythroid 2 like 2 (*Nrf2*), superoxide dismutase 1 (*SOD1*), glutathione peroxidase 1 (*GPX1*), and catalase (*CAT*). In addition, glyceraldehyde-3-phosphate dehydrogenase (*GAPDH*) was used as the reference gene in the current trial. All cDNA samples were assayed using quantitative real-time polymerase chain reaction (qPCR) amplifications by a StepOnePlus™ real-time PCR system (Applied Biosystems™, Waltham, MA, USA). The volume of the 15 μL reaction volume was as follows: 7.5 μL of 2 × SYBR Green qPCR master mix, 1.5 μL of 2.5 μM forward and reverse primers, 2.0 μL of cDNA, and 4.0 μL of ddH_2_O. The cycling conditions were as follows: 10 min at 95 °C for pre-denaturation, 40 cycles of 15 s at 95 °C for denaturation, and 30 s at 60 °C for extension.

### 2.9. Statistical Analysis

The sample size was calculated by Statistical Analysis System 9.1.3 software (SAS Institute, Cary, NC, USA). Each animal was treated as an experimental unit. All of the data analyses were performed using SAS 9.1.3 by the one-way ANOVA model: Y*_ij_* = µ + τ*_i_* + ε*_ij_*, where Y*_ij_* is the observation *j* (*j* = 1 to 6) in treatment *i*, µ is the overall mean, τ*_i_* is the effect of the treatment (denoted an unknown parameter), and ε*_ij_* is the random error with a mean of 0 and variance σ^2^ [25]. The significance level was set at *p* < 0.05.

## 3. Results

### 3.1. Growth Performance

No differences (*p* > 0.05) in DMI, BWC, ADG, or FCR were observed among the treatment groups, whereas the g/kg BW^0.75^ (%) of the two treatments was higher (*p* < 0.05) than that of the control (Table 4).

### 3.2. Meat Quality

The addition of PCP did not affect (*p* > 0.05) pH_45min_, pH_24h_, percentage of water loss, drip loss, cooking loss, or meat colour. However, the feeding of PCP could result in a significant (*p* < 0.05) decrease in muscle shear force value compared to the control group (Table 5).

### 3.3. Antioxidant Activity

The GSH, CAT, and DPPH scavenging activities in the HA group were greater (*p* < 0.05) than those in the control group (Table 6). The LA group showed higher (*p* < 0.05) POD activity than the other two groups. The feeding of 1.0 g/d of PCP exhibited higher (*p* < 0.05) O_2_^−^ and OH levels compared to the other groups.

### 3.4. Fatty Acid Profiles

Table 7 shows that the control group had higher (*p* < 0.05) levels of C12:0, C16:0, and total SFA than LA group. In terms of individual UFAs, C14:1, C16:1, C18:1n9 *cis*, C20:1, C18:2n6 *trans*, C18:3n3, C18:3n6, and C20:2 did not differ (*p* > 0.05) among all groups. However, the inclusion of PCP in goats resulted in an increase (*p* < 0.05) in individual UFAs for C18:1n9 *trans* and C20:3n6. Supplementation with 1.0 g/d of PCP led to an increase (*p* < 0.05) in the C20:4n6 level compared to the other two groups. The C18:2n6 *cis*, C20:3n6, C22:5n3, C22:6n3, and total PUFA levels were higher (*p* < 0.05) in goats that received 0.5 g/d PCP in comparison to the control group.

### 3.5. Gene Expression

**Figure 1:**
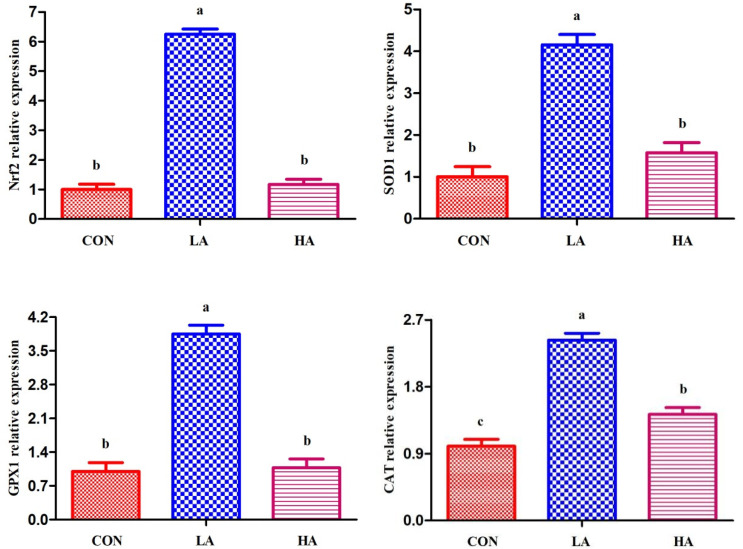
Relative mRNA abundance of genes of *longissimus thoracis et lumborum* muscle in goats. Data reported as least-squares means ± SEM (*n* = 6). Relative quantification of mRNA abundance for each gene was analysed by the 2^−∆∆Ct^ method, with the control group as the reference expression point. ^a–c^ Different letters indicate significant differences (*p* < 0.05). *Nrf2*, nuclear factor, erythroid 2 like 2; *SOD1*, superoxide dismutase 1; *GPX1*, glutathione peroxidase 1; *CAT*, catalase.

As shown in Figure 1, the abundance of Nrf2, SOD1, GPX1, and CAT was upregulated (*p* < 0.05) in the LTL of goats receiving 0.5 g/d PCP relative to the other groups. In addition, goats fed the control diet showed decreased CAT mRNA abundance (*p* < 0.05) compared to those fed the 0.5 g/d PCP diet.

## 4. Discussion

The DMI level is an important prerequisite for the growth performance of ruminants [26]. Anthocyanins may decrease DMI because of their bitter taste property, negatively affecting the performance of ruminants [27]. Our findings indicated that the intake of PCP did not affect DMI or performance in goats, suggesting that dietary supplementation with <1.0 g/d PCP anthocyanins has no side effects on animal performance under the experimental conditions. Similar results were observed in beef cattle after feeding anthocyanin-rich purple field corn stover, i.e., no differences in DMI and BW kg/d and g/kg BW^0.75^ [28].

Natural dietary antioxidant extracts may contain tenderising compounds, which can increase meat tenderness [29]. It has been demonstrated that meat tenderness is affected by muscle structural features [30]. Lipid oxidation in muscle is a chain reaction that involves FRs, producing low-molecular-weight volatile compounds and decreasing meat quality [31]. Thus, ROS could affect the turnover of intramuscular collagen in ruminant muscle, which means that OS status might impact meat tenderness [32]. In the current study, supplementation with anthocyanins resulted in a decrease in shear force, perhaps because anthocyanin may have enhanced muscle antioxidant activity, increasing meat tenderness in goats. In addition, anthocyanin is involved in lipid metabolism in animals, promoting adipose tissue deposition in muscles and, ultimately, improving muscle tenderness [33]. There is a protective effect of anthocyanins on proteolytic enzyme oxidation during ageing, which may also be an important reason for improving muscle tenderness [34]. Similar results were also observed with the inclusion of anthocyanin-rich plants in ruminant diets, which suggests that anthocyanin-rich plants do not affect meat pH, cooking loss, and drip loss, whereas they do reduce the shear force and increase instrumental meat tenderness [27].

The OS is the main cause of various metabolic diseases because it directly affects animal performance and causes a decline in the health of ruminants [35]. Indeed, OS is a condition involving an imbalance between oxidants and antioxidants, resulting in the production of abnormally high levels of FRs and a decline in antioxidant defence mechanisms in small ruminants [36]. Excessive FRs can react with DNA, proteins, and lipids, leading to DNA strand breakage and oxidative damage, protein–protein crosslinking, protein–DNA crosslinking, and lipid peroxidation, ultimately affecting body health [37]. Anthocyanins, as FR scavengers, can effectively complement the deficiencies of in vivo antioxidants, thereby playing a significant role in alleviating the OS status of ruminants [38]. Previous reports have suggested that the use of anthocyanin substances in ruminant feed could help prevent production loss due to OS [39,40]. Moreover, anthocyanins are potent natural antioxidants that can inhibit OS and inflammation by regulating peroxidation reactions and scavenging FRs in ruminants [23]. Thus, the results of the present study suggest that supplementation with anthocyanin-rich PCP could improve LTL antioxidant (GSH, CAT, and POD) status. The balance between oxidation and antioxidation systems in muscle tissue depends on antioxidant defence and FR levels, which are also important indicators of FA [41]. The PUFAs in muscle tissues might strengthen the body’s immune system, playing an important role in body health, whereas their double bonds are prone to oxidation. The feeding of 1.0 g/d PCP exhibited higher O_2_^−^ and OH concentrations in LTL, perhaps due to PUFAs being unable to tightly bind owing to the bending of the carbon chain in the presence of double bonds, leading to PUFAs with increased susceptibility to lipid oxidation. Moreover, goats receiving 1.0 g/d PCP may act as a “pro-oxidant” in the body, leading to high FR concentrations in muscle [10]. Our results were in agreement with those of Hosoda et al. [42], who previously demonstrated that sheep fed 0.5% anthocyanin-rich PCP in their diet exhibited suppression of oxidation resistance and enhanced plasma SOD content during 14-day periods. Moñino et al. [43] suggested that the addition of 10% phenolic-rich rosemary leaf extract to the feed of lamb for 240 days could increase the antioxidant status in meat. Similarly, Zhao et al. [34] showed that phenolic-rich grape pomace could increase TAC, SOD, and GPX4 and decrease ROS and MDA content in the LTL of lambs.

Ruminants have a high SFA content because of the extensive microbial biohydrogenation in the rumen. PUFAs are considered as being important compounds for consumers’ nutrition and health [44]. Vasta and Rui [45] suggested that decreased SFAs enhanced PUFAs in ruminant products. Additionally, Bryszak et al. [46] have shown that anthocyanin-rich plants could alter the biohydrogenation pathway, resulting in increased ruminal fluid *trans* C18:1 in dairy cows. Hence, supplementation of PCP in the basal diet for growing goats led to a decline in SFA content, perhaps because anthocyanins could shift the relative abundance of rumen microflora in goats [11]. Consistent with our results, Resconi et al. [47] found that lambs receiving phenolic-rich grape pomace showed decreased C16:0 and C17:0 levels in meat and increased C18:2n-6 content.

Lipid oxidation of meat includes three steps: FR initiation, reproduction, and termination. PUFAs oxidize lipid radicals, followed by the oxidation of peroxyl radicals with oxygen that then change FA hydroperoxide with hydrogen, thereby negatively affecting the body’s antioxidant enzymes [48]. Antioxidants can donate electrons to terminate the oxidation cycle at the propagation step, thus inhibiting the formation of additional lipid FRs and maintaining PUFA concentrations in meat [29]. A previous study showed that polyphenols could regulate rumen PUFA biohydrogenation to improve the lipid fraction by decreasing rumen skatole biosynthesis, enhancing FA content, and increasing the oxidation stability of the product [49]. Anthocyanins are water-soluble phenolic compounds that also prevent lipid oxidation. Indeed, anthocyanins have the ability to provide H-atoms to peroxyl radicals, thus inhibiting the oxidation of PUFAs by chain radical termination [50]. Flores et al. [51] suggested that the feeding of grape pomace silage in a lamb diet could increase the total PUFA content and maintain the stability of meat lipids and protein. In addition, anthocyanin may inhibit blood lipid mechanism parameters and modulate meat-related gene expression in goats [10]. As a consequence, we found that goats receiving anthocyanin-rich PCP could increase docosapentaenoate (DPA; C22:5n3), docosahexaenoate (DHA; C22:6n3), and total PUFA levels, which might be because anthocyanins have hydroxyl groups in the aromatic ring, which can provide extra hydrogen or electron donors and eliminate excessive FR in the body, thereby maintaining the PUFA concentration [50]. Moreover, SOD and CAT are involved in active oxygen scavenging and retain a balance of active oxygen metabolism, thereby inhibiting lipid oxidation in muscle. Thus, we found that PCP could enhance antioxidant activity in LTL, suggesting that these antioxidant enzymes can remove excessive FRs to maintain PUFA content, in turn retarding lipid oxidation in muscle. Consistent with our findings, Rana et al. [52] suggested that supplementation with phenolic-rich plant extracts led to increased PUFA concentrations in the LTL of crossbred kids. Bryszak et al. [46] also observed that supplementation of anthocyanin-rich plant additives to the daily ration of dairy cows could improve C18:2 *cis*-9, *trans*-11, C20:5n-3, DHA, and total PUFA levels in milk. Collectively, dietary supplementation with PCP anthocyanins could enhance the antioxidant activity and increase PUFA concentrations in muscle, subsequently improving meat quality of goats.

*Nrf2* is a sensor protein of xenobiotic toxic substances and OS because it plays an important role in participating in cellular antioxidant stress and the major defence mechanism induced by xenobiotic toxic substances [53]. Various studies have reported that the activation of the Nrf2/antioxidant responsive element signalling pathway can induce an endogenous increase in antioxidant enzymes, antioxidant proteins, and anti-inflammatory and detoxification proteins, all of which can play a protective role [54,55]. Anthocyanins are *Nrf2*-activating agents that can directly modify sensor cysteines present in kelch-like ECH protein (Keap1), thereby activating *Nrf2* and regulating the expression of cytoprotective proteins with anti-inflammatory and antioxidant functions [56]. Indeed, anthocyanins may directly interact with cysteine residues present in Keap1, thereby stimulating *Nrf2* dissociation and phase II detoxification or antioxidant enzymes, such as SOD, GPX, and CAT [57]. Liu et al. [58] demonstrated that mulberry anthocyanin extract could improve the expression levels of the downstream antioxidant target genes in rat liver by activating the *Nrf2* signalling pathway. As a result, the feeding of 0.5 g/d PCP can increase *Nrf2* expression, thereby increasing the expression of *SOD1*, *GPX1*, and *CAT* in the LTL of goats. Consistent with our results, Tian et al. [16] showed that anthocyanin can increase the abundances of *Nrf2* and *GPX1* genes in the mammary gland of Saanen dairy goats. Of interest, a high dose of anthocyanins might have side effects on the body by acting as a “pro-oxidant” that generates toxic oxygen [59]. Hence, goats receiving 1.0 g/d PCP did not exhibit improved antioxidant gene expression, except for CAT in the LTL compared to the control.

## 5. Conclusions

The results of the current study indicate that PCP can be used as a source of natural functional additive for growing goats, because (1) PCP anthocyanins can improve meat quality, (2) PCP anthocyanins has the potential to enhance muscle antioxidant status and regulate muscle related antioxidant gene expression, and (3) PCP anthocyanins can improve muscle PUFA concentrations. However, the underlying mechanisms require further investigation. Studies on the bioavailability of anthocyanin in the body that elucidate the mechanism by which anthocyanin improves the antioxidant status and prevents lipid oxidation of ruminants are warranted.

## Figures and Tables

**Table 1:** Anthocyanin composition of the purple corn pigment.

Items, µg/g DM	Content
Pelargonidin	45.3 ± 1.9
Peonidin	ND
Cyanidin	1975 ± 8.4
Malvidin	0.1 ± 0.01
Petunidin	7.9 ± 0.1
Delphinidin	591 ± 11.9
Total anthocyanins	2619 ± 13.04

Values represent the mean of three replicates (*n* = 3). Values are means ± standard deviation; ND = not detected.

**Table 2:** Ingredients and nutrient composition of basal diet.

Ingredients (g/kg Fed Basis)	Content	Chemical Composition (g/kg of DM)	Content
Peanut vines	500	Dry matter (g/kg of the as-fed diet)	902
White distiller’s grains	100	Crude protein	138
Soybean residues	100	Gross energy, kJ/g	133
Green hay	93	Neutral detergent fibre	435
Corn	140	Acid detergent fibre	292
Soybean meal	50	Hemicellulose	143
Mineral premix	5	Ether extract	22.7
Vitamin premix	5	Organic matter	915
NaCl	5		
Limestone	2		
Total	1000		

Treatment 1 and treatment 2 were given a basal diet with 0.5 g/d and 1.0 g/d purple corn pigment, respectively. Vitamin premix was purchased from Guangzhou Everrich Animal Health Co., Ltd. (Guangzhou, China), containing, per kg: 4,000,000 IU vitamin A; 600,000 IU vitamin D; 25,000 mg vitamin E; 7000 mg dl-methionine; 5000 mg l-lysine. Mineral premix was obtained from the Earth Animal Nutrition and Health Products Co., Ltd. (Chongqing, China), containing, per kg: 1300 mg Cu; 1000 mg Fe; 1575 mg Zn; 595 mg Mn.

**Table 3:** Primer sequences used for real-time PCR amplifications in this study.

Gene	Primer Sequences (5′ to 3′)	Accession Number	Product Size (nt)
*Nrf2*	(F) GTTCGTTCAGGTAGCCACTGCT	NM_001314327.1	154
	(R) TTTGGTTTCTGGACTTGGAACTGTA		
*SOD1*	(F) GGCAAAGGGAGATAAAGTCGTC	NM_001285550.1	197
	(R) CCTTCACATTGCCCAGGTCTC		
*GPX1*	(F) GCATCGCTCTGAGGCACAAC	XM_005695962.3	134
	(R) TCGTTCTTGGCATTTTCCTGA		
*CAT*	(F) CCAGCGACCAGATGAAACACT	XM_005690077.3	277
	(R) AACACCTTCGCCTTGGAGTATC		
*GAPDH*	(F) GCCCTCTCAAGGGCATTCTA	XM_005680968.3	81
	(R) AGGTAGAAGAGTGAGTGTCGC		

*Nrf2*, nuclear factor, erythroid 2 like 2; *SOD1*, superoxide dismutase 1; *GPX1*, glutathione peroxidase 1; *CAT*, catalase; *GAPDH*, glyceraldehyde-3-phosphate dehydrogenase; F, forward; R, reverse.

**Table 4:** Effect of purple corn pigment on DMI and growth performance of goats.

Items	Group			SEM	*p*-Value
	Control	0.5 g/d of PCP	1.0 g/d of PCP		
DMI, g/d	702	715	721	6.523	0.12
g/kg BW^0.75^, %	69.6 ^b^	72.0 ^a^	73.4 ^a^	0.655	0.0002
Initial weight, kg	21.8	21.3	21.0	0.583	0.64
Final weight, kg	25.2	24.6	24.5	0.582	0.67
BWC, kg	3.38	3.24	3.49	0.161	0.57
ADG, g	56.3	54.1	58.1	2.685	0.58
FCR	12.7	13.3	12.6	0.604	0.67

Means in the same row with no superscript letters after them or with a common superscript letter following them are not significantly different at *p* < 0.05. Values represent the mean of six replicates (*n* = 6). SEM, standard error of mean; DMI, dry matter intake; BW, body weight (g/kg BW^0.75^, % was % of DMI per kg of metabolic BW, respectively); BWC, body weight change; ADG, average daily gain; FCR, feed conversion ratio.

**Table 5:** Effect of purple corn pigment on *longissimus thoracis et lumborum* muscle quality of goats.

Items		Group		SEM	*p*-Value
	Control	0.5 g/d of PCP	1.0 g/d of PCP		
pH_45min_	5.58	6.03	5.55	0.208	0.22
pH_24h_	5.14	5.36	4.98	0.296	0.68
Percentage of water loss, %	4.61	4.39	4.41	0.590	0.96
Drip loss, %	1.30	1.05	1.36	0.241	0.71
Cooking loss, %	68.8	65.9	65.7	1.408	0.24
Meat colour (L*), Opto	55.3	58.3	52.9	3.766	0.60
Shear force, kg	5.31 ^a^	4.24 ^b^	4.61 ^b^	0.201	0.006

Means in the same row with no superscript letters after them or with a common superscript letter following them are not significantly different at *p* < 0.05. Values represent the mean of six replicates (*n* = 6). SEM, standard error of mean.

**Table 6:** Effect of purple corn pigment on *longissimus thoracis et lumborum* muscle antioxidant activity of goats.

Items		Group		SEM	*p*-Value
	Control	0.5 g/d of PCP	1.0 g/d of PCP		
TAC, U/mL	14.9	12.2	15.0	1.509	0.39
SOD, U/mL	23.0	22.5	22.4	0.379	0.52
GPX, U/mL	127	131	140	5.507	0.29
GSH, mg/L	14.5 ^b^	20.4 ^b^	27.7 ^a^	2.223	0.008
CAT, U/mL	14.3 ^b^	18.7 ^a^	19.0 ^a^	0.424	<0.0001
POD, U/mg	4.61 ^b^	6.69 ^a^	5.31 ^b^	0.311	0.003
MDA, nmol/mL	2.59	2.93	2.12	0.332	0.30
NO, μmol/L	42.2	38.2	30.5	4.714	0.28
DPPH scavenging activity, %	4.39 ^b^	6.17 ^a,b^	11.7 ^a^	1.647	0.047
O_2_^−^, U/L	185 ^b^	197 ^a^	196 ^a^	2.403	0.003
OH, U/mL	81.8 ^a,b^	80.2 ^b^	83.4 ^a^	0.629	0.009

Means in the same row with no superscript letters after them or with a common superscript letter following them are not significantly different at *p* < 0.05. Values represent the mean of six replicates (*n* = 6). SEM, standard error of mean; TAC, total antioxidant capacity; SOD, superoxide dismutase; GPX, glutathione peroxidase; GSH, reduced glutathione; CAT, catalase; POD, peroxidase; MDA, malondialdehyde; DPPH, 2,2-diphenyl-1-picrylhydrazyl; NO, nitric oxide; O_2_^−^, superoxide anion; OH, hydroxyl free radical.

**Table 7:** Effect of purple corn pigment on fatty acid profile (g/100 g of total fatty acid) the *longissimus thoracis et lumborum* muscle of goats.

Items	Group			SEM	*p*-Value
	Control	0.5 g/d of PCP	1.0 g/d of PCP		
Laurate (C12:0)	0.043 ^a^	0.028 ^b^	0.037 ^a,b^	0.003	0.033
Myristate (C14:0)	0.84	0.71	0.81	0.048	0.28
Pentadecanoate (C15:0)	0.087	0.068	0.085	0.006	0.17
Palmitate (C16:0)	28.9 ^a^	25.1 ^b^	26.7 ^a,b^	0.582	0.044
Stearate (C18:0)	22.1	20.8	21.2	0.397	0.22
Arachidate (C20:0)	0.068	0.072	0.065	0.010	0.88
Behenate (C22:0)	0.097	0.062	0.075	0.011	0.21
Total SFA	52.1 ^a^	46.9 ^b^	49.0 ^a,b^	0.863	0.042
Myristoleate (C14:1)	0.15	0.16	0.20	0.022	0.37
Palmitoleate (C16:1)	2.97	3.13	3.36	0.202	0.48
Elaidate (C18:1n9 *trans*)	0.14 ^b^	0.19 ^a^	0.18 ^a^	0.006	0.021
Oleate (C18:1n9 *cis*)	35.3	36.7	35.4	0.890	0.53
11-Eicosenoate (C20:1)	0.058	0.061	0.061	0.009	0.96
Total MUFA	38.6	40.2	39.2	0.853	0.47
Linoelaidate (C18:2n6 *trans*)	0.057	0.053	0.062	0.011	0.86
Linoleate (C18:2n6 *cis*)	7.21 ^b^	10.89 ^a^	9.11 ^a,b^	0.535	0.039
Alpha linolenate (C18:3n3)	0.69	0.61	0.73	0.045	0.30
Gamma linolenate (C18:3n6)	0.26	0.28	0.27	0.014	0.60
11-14 Eicosadienoate (C20:2)	0.11	0.13	0.15	0.013	0.28
Homo-gamma linolenate (C20:3n6)	0.13 ^b^	0.19 ^a^	0.19 ^a^	0.005	0.009
Arachidonate (C20:4n6)	0.68 ^b^	0.64 ^b^	1.00 ^a^	0.044	0.017
Docosapentaenoate (DPA; C22:5n3)	0.034 ^c^	0.102 ^a^	0.062 ^b^	0.005	0.006
Docosahexaenoate (DHA; C22:6n3)	0.048 ^b^	0.096 ^a^	0.061 ^b^	0.006	0.027
Total PUFA	9.27 ^b^	12.87 ^a^	11.70 ^a,b^	0.628	0.038

Means in the same row with no superscript letters after them or with a common superscript letter following them are not significantly different at *p* < 0.05. Values represent the mean of six replicates (*n* = 6). SEM, standard error of mean; ND, not detected; SFA, sum of all the saturated fatty acid; MUFA, sum of all the monounsaturated fatty acid; PUFA, sum of all the polyunsaturated fatty acid; SFA, sum of all the saturated fatty acid. SFA: all saturated fatty acids from C12:0 to C22:0 without any double bond; MUFA: all monounsaturated fatty acids from C14:1 to C20:1 with single double bond; PUFA: all polyunsaturated fatty from acids from C18:2n6 *trans* to C22:6n3 with two or more double bonds.

## Data Availability

The data presented in the current study are available on request from the corresponding author.
